# Trends in the incidence and mortality of colorectal cancer in a brazilian city

**DOI:** 10.1186/s13104-020-05411-9

**Published:** 2020-12-09

**Authors:** Alex Rodrigues Moura, Adriane Dórea Marques, Mylena Santos Dantas, Érika de Abreu Costa Brito, Mariana do Rosário Souza, Marcela Sampaio Lima, Hianga Fayssa Fernandes Siqueira, Angela Maria da Silva, Ana Carolina Ribeiro Lisboa, Marco Antonio Prado Nunes, Marceli de Oliveira Santos, Carlos Anselmo Lima

**Affiliations:** 1 Aracaju Cancer Registry, Aracaju, Sergipe Brazil; 2 Health Sciences Graduate Program, Aracaju, Sergipe Brazil; 3 University Hospital, EBSERH/Federal University of Sergipe, Aracaju, Sergipe Brazil; 4 CONPREV/Brazilian National Cancer Institute, Rio de Janeiro, Brazil

**Keywords:** Colorectal cancer, Demography, Incidence, Mortality

## Abstract

**Objectives:**

This study was conducted to analyze the trends in colorectal cancer (CRC) incidence and mortality in the city of Aracaju, Sergipe State, Brazil, between 1996 and 2015 with Joinpoint Regression Program 4.7.0.0 and to identify the geographical distribution of CRC in the municipality.

**Results:**

A total of 1322 cases of CRC and 467 CRC-related deaths during the study period were included. In total, 40% of the incident cases and 43% of the deaths occurred in men, while 60% of the incident cases and 57% of the deaths occurred in women. Males who were 20 to 44 years old had the most significant trend in growth. Among women, those in the group aged 45 to 64 years had the highest observed annual percent change (APC). In both sexes, mortality was stable. Regarding the geographic distribution, there were constant hotspots in the northeast region of the municipality. This study showed a significant increase in incidence, mainly in young men between 20 and 44 years of age, but stable mortality in Aracaju.

## Introduction

The epidemiological transition occurring throughout the world and in Brazil has drawn the attention of the health care field towards noncommunicable chronic diseases (NCDs) [1]. Cardiovascular diseases, respiratory diseases, diabetes and its complications and cancer are pathologies that contribute to the increased incidence of and mortality due to NCDs.

The incidence of and mortality due to colorectal cancer (CRC) has increased in several regions of the world [2]. In the United States, in 2018, it was the fourth most common type of cancer diagnosed in both sexes [3, 4]. In Brazil, estimates calculated by the José de Alencar Gomes da Silva National Cancer Institute (INCA) for the 2020–2022 triennium showed that when nonmelanoma tumors were excluded, CRC ranked between the second and fourth leading cancer in men and between the second and third leading cancer in women, depending on the studied region [5].

Given its features, such as the high possibility of prevention by screening examinations and the high cure rate if diagnosed in the early stages, a study of the trends in the CRC incidence and mortality rates in the city of Aracaju, the capital of Sergipe State, Brazil, located at 10° 54′ 36″ S, 37° 4′ 12″ W, which has a Human Development Index (HDI) of 0.770 and an estimated population in 2020 of 664,908 inhabitants, is essential [6, 7]. Assessments of the topographical distribution, proportions of histological subtypes, mortality/incidence ratio and spatial distribution of this disease are also extremely valuable for understanding the state of this disease and making decisions about epidemiological control measures.

## Main text

### Methods

The present study was a partially ecological study of time series aggregates divided into three parts: descriptive analysis, trend analysis and geoprocessing.

The included patients were men and women who were diagnosed with and/or died due to CRC from 1996 to 2015 and were registered in the Population-Based Cancer Registry (RCBP) and/or in the Mortality Information Service (SIM) under the following International Classification of Diseases in Oncology, 2nd and 3rd edition codes: C18 to C20. Regarding morphology, the codes were 80003, 80103, 80203, 80513, 80703, 80723, 81233, 81243, 81403, 82013, 82103, 82113, 82203, 82403, 82463, 82603, 82613, 82623, 82633, 84803, 84813, 84903, 85603, 88003, 89363, 95903, 95913, 96823, 96873, 96923, 96983, 96993, and 97153. These data were collected according to the International Agency for Research on Cancer (IARC) standards [8, 9] and were validated in Brazil by the INCA.

For the descriptive analysis, the following data were calculated for each sex and for both sexes combined: the rates of incident cases and mortality, proportions of histological types, topographic distribution of the disease in the colon and the mortality/incidence ratio [10]. To calculate the rates, the half-period population for each age group provided by the Brazilian Institute of Geography and Statistics (*Instituto Brasileiro de Geografia e Estatística*–IBGE) [11] was used as the basis, with age standardization to the global population [12]. An additional file shows this in more detail (see Additional file 1).

The trends in incidence and mortality were calculated using Joinpoint Regression Program 4.7.0.0. This model was chosen because it facilitates the analysis of temporal trends and the assessment of whether there are changes in the observed trend at specific points (joinpoints). It identifies annual percent changes (APCs) and their averages (AAPCs) over the study period. The software calculates the trends, starting with the minimum joinpoint of 0, and tests the statistical significance (Monte Carlo test) of the changes after adding more joinpoints [10]. To examine the trends in mortality, the age groups of 45 to 64 years, older than 65 years and all ages were selected, given that the incidence and, consequently, mortality are much lower among younger individuals and that several years had no reported deaths in younger age groups, making it impossible to calculate the trends.

The spatial distribution of the density of incident CRC cases was mapped using the heat map function (kernel function) of the Geographic Information System (GIS) multiplatform software QGIS 2.18.0 [13]. Address coordinates were collected by the RCBP, and inconsistent addresses were individually evaluated and obtained using Google Maps.

## Results

Between 1996 and 2015, 1322 cases of CRC were registered in the RCBP, with 40.5% in males and 59.5% in females. The SIM recorded 467 deaths in the same period; 42.6% of those who died were males and 57.4% were females (Table 1). Regarding the topographic distribution of CDC, in both sexes, the left colon and rectum were the most common sites (Table 1). Among the histological subtypes, adenocarcinoma was the most common (Table 1).

**Table 1:** Incident cases and deaths, the mortality/incidence ratio, the topographic distribution and the proportions of histological subtypes stratified by sex

	Male	Female
Total cases	535	787
Deaths	199	268
Incident cases and mortality	40.5; 42.6%	59.5; 57.4%
Topographic distribution		
Right	12.5%	13.72%
Transverse	2.99%	3.43%
Left	28.4%	28%
Colon (not specified)	24.11%	21.98%
Overlapping^a^	4.48%	1.9%
Rectum	25.79%	28%
Appendix	1.68%	2.79%
Histological subtypes		
Adenocarcinoma	95%	93.69%
Lymphoma	0.93%	0.76%
N. endocrine	3.17%	5.2%
Sarcoma	0.37%	0.38%

^a^Overlapping sites

**Fig. 1:**
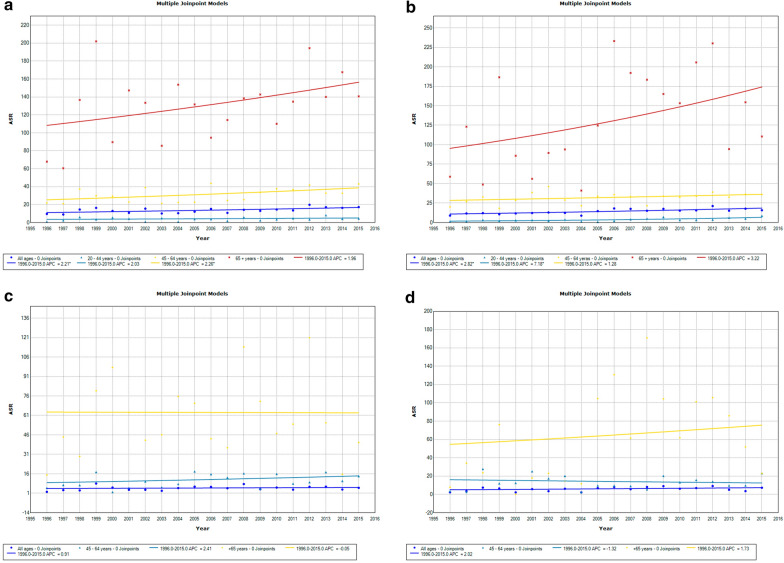
Incidence and mortality trends: **a**, **b** Incidence in females and males, respectively, considering the adjusted rates for the age groups 20–44 years, 45–64 years, + 65 years and all ages; **c**, **d** mortality in females and males, respectively, considering the adjusted rates for the age groups 45–64 years, + 65 years and all ages

The analysis of the incidence showed constant growth in both sexes; however, the mortality trends in all age groups were stable (Fig. 1). In terms of the incidence, the highest APC was observed in women between 45 and 64 years old, with an APC of 2.3 (95% CI 0.4–4.2). Among men, the highest APC of 7.2 (95% CI 3.7–10.8) was identified in the group aged 20 to 44 years.

There were no significant trends in mortality in women and men in the specified age groups (45 to 64 years, + 65 years and all ages).

**Fig. 2:**
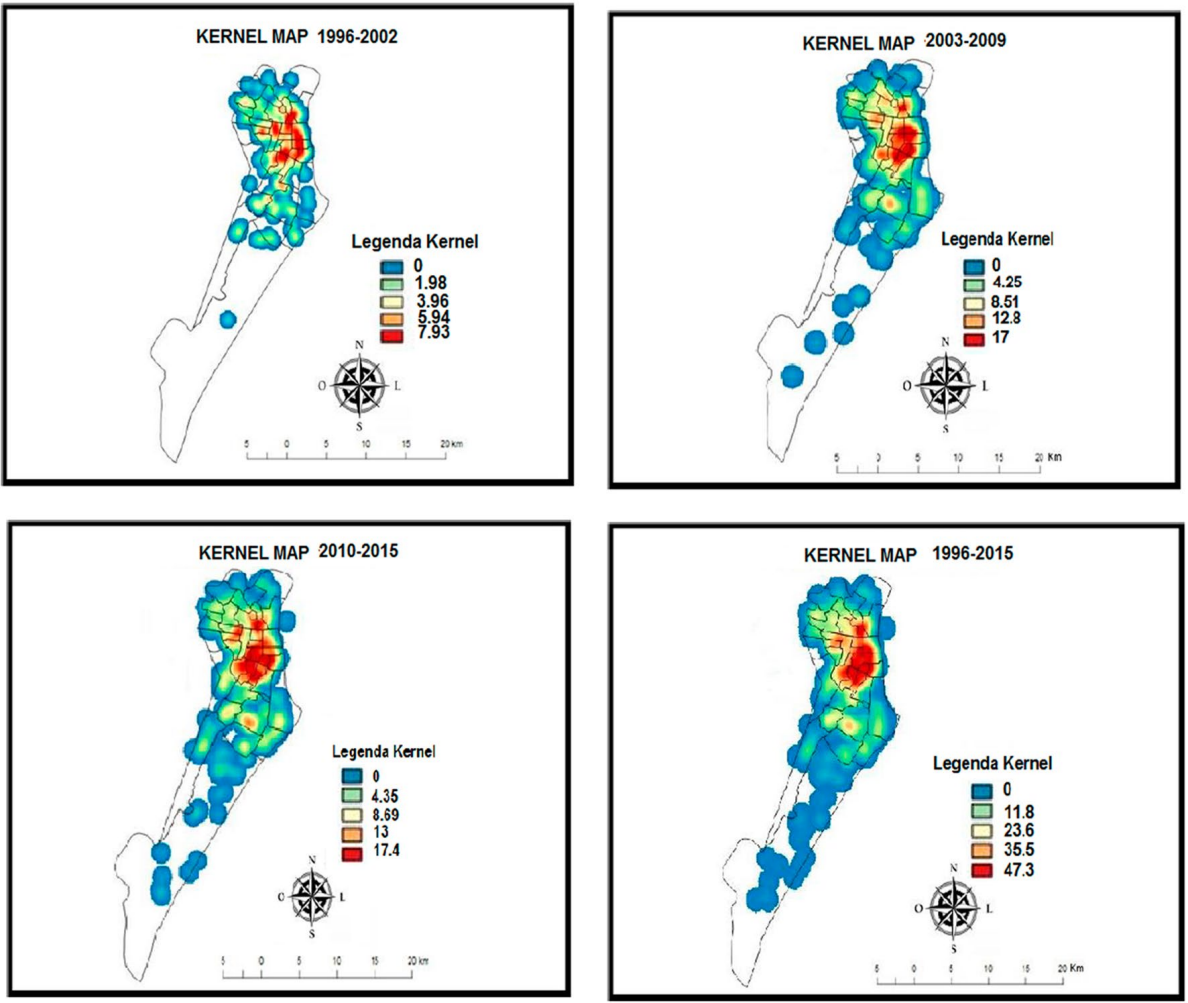
Evolution of the density of incident cases of CRC on the kernel map

The kernel maps covered three periods, with an additional map showing the entire study period (1996–2002, 2003–2009, 2010–2015 and 1996–2015) (Fig. 2). In the first period, the areas with the highest density were the central and northeast regions, followed by small scattered points in the north and central-south regions. In the second period, the density in the northeast of the municipality was maintained, while that in the central region increased. In the third analysis period, the highest density was still in the northeast region, as in the second study period.

## Discussion

When the trends in incidence trends were evaluated by age groups based on life stages (0–19, 20–44, 45–64 and + 65 years), there was a stable and constant increase in incidence in both females and males. Among women, the APCs had little variation. Among men, the highest APC was observed among the youngest group (20 to 44 years). As CRC has a multifactorial etiology, factors such as an increase in weight secondary to changes in dietary patterns and a sedentary lifestyle may contribute to this increase [14]. In countries with a very high HDI, such as the United States, a decrease in the incidence rate of CRC among individuals older than 50 years has been observed, while other groups have increasing or constant APCs of 2.7 (< 40 years) and 1.7 (40 to 49 years) [15]. The decrease in the incidence of CRC in individuals over 50 years is most likely due to public health policies [15]. Because CRC is largely preventable, screenings reduce the incidence among the elderly population; however, as screening is performed earlier, this results in an increase in the incidence rate in the younger population [16].

Regarding mortality, the trends among the groups of men and women over 65 years, between 45 and 64 years and all ages were stable. Studies have shown that in regions with an HDI similar to Brazil, the trends in CRC incidence and mortality are increasing [17]. This reflects the difficulty of access to healthcare, with consequent advanced stages at diagnosis [18, 19], in addition to poor infrastructure, the lack of adequate screening and treatment [20], and the presence of age-related comorbidities [21].

During the study period, women had more incident cases and deaths than men (59.5% and 40.5%; 57.4 and 42.6%, respectively). This finding differs from the results obtained by Ansa et al. [15], who evaluated data from the Surveillance, Epidemiology, and End Results (SEER) Program and observed that CRC was more prevalent among men from 2000 to 2014 in the United States. This difference may be due to the different male: female ratios in the two study areas [22, 23]. In the state of Sergipe, according to the 2010 census, among individuals older than 40 years, the male:female ratio was 1:1.16, while in the United States, the ratio for the same period was 1:1.09 [22, 23].

When the adjusted incidence rates were analyzed, we observed that females and males had similar intermediate levels of variation. These intermediate values occurred due to the epidemiological transition observed in countries with a lower HDI. The highest incidence rates are concentrated in regions with higher HDIs, such as Australia and New Zealand (36.7 cases per 100,000), Europe (28.8–32.1 cases per 100,000), East Asia (26.5 cases per 100,000) and North America (26.2 cases per 100,000) [24]. In regions with lower HDIs, such as Africa and South-Central Asia, the incidence rates are lower (6.4–9.2 per 100,000 and 4.9 per 100,000, respectively) [24].

For both sexes, the left colon and rectum were the most common sites of CRC (Table 1). The epidemiological relevance of these data results from the fact that the most common sites are accessible by flexible rectosigmoidoscopy, which may be a less expensive and more effective screening measure in economically and socially less-developed countries. Ahnen et al. [25] observed a higher prevalence of CRC in the left colon and rectum in the US in 2014, especially among younger individuals.

When the histological subtypes of CRC were considered, approximately 94.5% of the CRCs were adenocarcinoma in both sexes. These values are similar to those described in the international and US literature, in which more than 90% of CRCs are adenocarcinomas [26].

The kernel map showed the geographic distribution, with a hotspot in the northeastern region. This result may be due to the higher population in that region, according to the 2010 census [27]. When analyzing the hotspots according to the 2000 census, there was a small change in the population density [27]. According to the monthly household income distribution by neighborhood based on the 2000 census data, the neighborhoods with the highest monthly household income levels are in the hotspots for the entire study period (1996–2015) [27, 28]. This corroborates the relationship between a higher HDI and a higher incidence of CRC [29]. The neighborhoods with the lowest monthly incomes were also located in the hotspots due to their proximity to the more affluent areas and their population density.

The strengths of our study were as follows: a long study period (1996–2015) and incidence data that were validated nationally and internationally. The database was of high quality, with 94.8% of the diagnoses verified with pathology, and only 3.5% based on death certificates and 1.6% based on C80.

## Conclusion

The incidence rates of CRC have been increasing steadily in the municipality of Aracaju and are similar to those in regions with similar HDIs. The APC among young men is noteworthy, showing that the incidence of CRC is following the same pattern noted in countries with higher HDIs. Mortality was stable in both sexes. Regarding topography, the left colon and the rectum were the most common site. Adenocarcinoma was the most common histological subtype. Our findings indicate that public health measures are needed. Exams such as flexible rectosigmoidoscopy every 5 years and annual fecal occult blood tests are relatively inexpensive and, according to the literature, will reduce the rates of the third most common malignant neoplasia worldwide.

## Limitations

The limitations were the difficulty of obtaining geographic coordinates, mainly in the earliest period. However, only 7% of the data were lost. For the mortality calculations, the collected data were released by the SIM. It is known that the completion of death certificates depends on the declarant, which can affect the conclusions due to underestimation. However, the data we used were official. Improvements in filling out death certificates regarding cancer are ongoing and are critical for accurate monitoring [30].

## Supplementary Information

**Additional file 1.** Present raw date on how taxes were calculated.

## Data Availability

The datasets used and/or analyzed during the current study are available from the corresponding author on reasonable request.

## Abbreviations

APC: Annual percent change
AAPC: Annual average percentage change
CAAE: Certificate of Presentation of Ethical Appreciation
GIS: Geographic information system
HDI: Human Development Index
IARC: International Agency for Research on Cancer
INCA: Cancer National Institute
CRC: Colorectal cancer
RCBP: Population-based cancer registry
SIM: Mortality Information Service
UFS: Federal University of Sergipe
