# Supplementary material for: Trends in the incidence and mortality of colorectal cancer in a brazilian city
Source: BMC Res Notes. 2020 Dec 9;13:560. doi: 10.1186/s13104-020-05411-9 (PMC7727142; doi:10.1186/s13104-020-05411-9)
Supplement: Supplementary file 1 — Additional file 1. Present raw date on how taxes were calculated. [file 13104_2020_5411_MOESM1_ESM.pdf]

1) Table. Adjusted incidence and mortality in adenocarcinoma rates for both sexes per 100,000.

	INCIDENCE				MORTALITY			
YEAR	ASR F	95% CI	ASR M	95% CI	ASR F	95% CI	ASR M	95% CI
1996	9.6	4.7; 14.4	8.9	3.4; 14.4	2.07	-0.27; 4.42	2.29	-0.30; 4.87
1997	9.0	4.4; 13.5	11.7	5.6; 17.9	3.44	0.89; 5.99	3.63	0.07; 7.18
1998	14.5	9.2; 19.8	12.1	5.8; 18.4	3.23	0.84; 5.62	7.43	2.57; 12.28
1999	16.3	10.6; 22.1	10.6	5.0; 16.2	8.47	4.19; 12.76	6.47	1.99; 10.95
2000	12.9	8.0; 17.7	11.7	5.8; 17.6	5.36	2.44; 8.27	2.19	-0.29; 4.68
2001	10.8	6.5; 15	12.2	6.7; 17.8	3.67	1.27; 6.07	5.83	1.51; 10.15
2002	15.5	10.2; 20.8	12.9	6.9; 18.8	3.74	1.15; 6.33	3.47	0.43; 6.51
2003	10.1	6.1; 14.2	12.4	6.7; 18.1	2.90	0.75; 5.04	6.20	2.36; 10.04
2004	10.4	6.5; 14.4	8.8	4.2; 13.4	5.04	2.30; 7.78	2.23	-0.29; 4.76
2005	12.1	7.9; 16.3	14.7	8.5; 20.8	6.09	3.20; 8.99	6.93	2.84; 11.03
2006	15.2	10.4; 19.9	18.1	11.7; 24.4	5.88	2.80; 8.96	8.08	3.51; 12.65
2007	10.8	7.0; 14.6	17.6	11.4; 23.8	4.93	2.35; 7.51	5.69	2.17; 9.22
2008	14.2	9.8; 18.5	15.1	9.4; 20.7	8.15	4.82; 11.48	8.15	3.88; 12.42
2009	13.0	9.0; 16.9	17.6	11.7; 23.5	4.14	1.89; 6.4	8.95	4.56; 13.34
2010	14.5	10.3; 18.6	15.3	10.0; 20.6	5.40	2.91; 7.9	6.22	2.84; 9.6
2011	13.5	9.5; 17.4	15.6	10.2; 21	3.78	1.73; 5.84	6.88	3.28; 10.48
2012	19.8	15.0; 24.6	21.2	15.0; 27.4	6.02	3.51; 8.54	9.24	5.08; 13.39
2013	17.1	12.8; 21.5	15.2	10.2; 20.2	6.12	3.44; 8.81	5.09	2.08; 8.1
2014	16.3	12.1; 20.5	17.8	12.4; 23.3	3.94	1.88; 6.01	3.60	1.25; 5.96
2015	17.2	12.9; 21.6	15.8	11.0; 20.6	5.17	2.78; 7.56	7.35	3.95; 10.74

Legend: ASR: age-standardized rate; F: females; M: males.

2) Table. Trends incidence female CRC

Annual Percent Change (APC)								
Cohort	Segment	Lower Endpoint	Upper Endpoint	APC	Lower CI	Upper CI	Test Statistic (t)	Prob > |t|
All ages - 0 Joinpoints	1	1996	2015	2.2*	0.8	3.7	3.3	0.0
20 - 44 years - 0 Joinpoints	1	1996	2015	2.0	-1.9	6.1	1.1	0.3
45 - 64 years - 0 Joinpoints	1	1996	2015	2.3*	0.4	4.2	2.6	0.0
65 + years - 0 Joinpoints	1	1996	2015	2.0	-0.2	4.1	1.9	0.1

* Indicates that the Annual Percent Change (APC) is significantly different from zero at the alpha = 0.05 level.

Average Annual Percent Change (AAPC)								
Cohort	Range	Lower Endpoint	Upper Endpoint	AAPC	Lower CI	Upper CI	Test Statistic~	P-Value~
All ages - 0 Joinpoints	Full Range	1996	2015	2.2*	0.8	3.7	3.3	0.0
20 - 44 years - 0 Joinpoints	Full Range	1996	2015	2.0	-1.9	6.1	1.1	0.3
45 - 64 years - 0 Joinpoints	Full Range	1996	2015	2.3*	0.4	4.2	2.6	0.0
65 + years - 0 Joinpoints	Full Range	1996	2015	2.0	-0.2	4.1	1.9	0.1

* Indicates that the AAPC is significantly different from zero at the alpha = 0.05 level.
~ If the AAPC is within one segment, the t-distribution is used. Otherwise, the normal (z) distribution is used. See Help to Learn More

3) Table. Trends incidence male CRC

Annual Percent Change (APC)								
Cohort	Segment	Lower Endpoint	Upper Endpoint	APC	Lower CI	Upper CI	Test Statistic (t)	Prob > |t|
All ages - 0 Joinpoints	1	1996	2015	2.8*	1.5	4.2	4.4	0.0
20 - 44 years - 0 Joinpoints	1	1996	2015	7.2*	3.7	10.8	4.4	0.0
45 - 64 veras - 0 Joinpoints	1	1996	2015	1.3	-0.4	3.0	1.6	0.1
65 + years - 0 Joinpoints	1	1996	2015	3.2	-0.6	7.2	1.8	0.1

* Indicates that the Annual Percent Change (APC) is significantly different from zero at the alpha = 0.05 level.

Average Annual Percent Change (AAPC)								
Cohort	Range	Lower Endpoint	Upper Endpoint	AAPC	Lower CI	Upper CI	Test Statistic~	P-Value~
All ages - 0 Joinpoints	Full Range	1996	2015	2.8*	1.5	4.2	4.4	0.0
20 - 44 years - 0 Joinpoints	Full Range	1996	2015	7.2*	3.7	10.8	4.4	0.0
45 - 64 veras - 0 Joinpoints	Full Range	1996	2015	1.3	-0.4	3.0	1.6	0.1
65 + years - 0 Joinpoints	Full Range	1996	2015	3.2	-0.6	7.2	1.8	0.1

* Indicates that the AAPC is significantly different from zero at the alpha = 0.05 level.
~ If the AAPC is within one segment, the t-distribution is used. Otherwise, the normal (z) distribution is used. See Help to Learn More

4)

Table. Trends mortality female CRC

Annual Percent Change (APC)								
Cohort	Segment	Lower Endpoint	Upper Endpoint	APC	Lower CI	Upper CI	Test Statistic (t)	Prob > |t|
All ages - 0 Joinpoints	1	1996	2015	0.9	-1.8	3.6	0.7	0.5
45 - 64 years - 0 Joinpoints	1	1996	2015	2.4	-1.5	6.5	1.3	0.2
+65 years - 0 Joinpoints	1	1996	2015	-0.1	-4.5	4.6	-0.0	1.0

* Indicates that the Annual Percent Change (APC) is significantly different from zero at the alpha = 0.05 level.

Average Annual Percent Change (AAPC)								
Cohort	Range	Lower Endpoint	Upper Endpoint	AAPC	Lower CI	Upper CI	Test Statistic~	P-Value~
All ages - 0 Joinpoints	Full Range	1996	2015	0.9	-1.8	3.6	0.7	0.5
45 - 64 years - 0 Joinpoints	Full Range	1996	2015	2.4	-1.5	6.5	1.3	0.2
+65 years - 0 Joinpoints	Full Range	1996	2015	-0.1	-4.5	4.6	-0.0	1.0

* Indicates that the AAPC is significantly different from zero at the alpha = 0.05 level.
~ If the AAPC is within one segment, the t-distribution is used. Otherwise, the normal (z) distribution is used. See Help to Learn More

5) Table. Trends mortality male CRC

Annual Percent Change (APC)								
Cohort	Segment	Lower Endpoint	Upper Endpoint	APC	Lower CI	Upper CI	Test Statistic (t)	Prob > |t|
All ages - 0 Joinpoints	1	1996	2015	2.0	-1.0	5.2	1.4	0.2
45 - 64 years - 0 Joinpoints	1	1996	2015	-1.3	-5.7	3.2	-0.6	0.5
+65 years - 0 Joinpoints	1	1996	2015	1.7	-5.9	10.0	0.5	0.7

* Indicates that the Annual Percent Change (APC) is significantly different from zero at the alpha = 0.05 level.

Average Annual Percent Change (AAPC)								
Cohort	Range	Lower Endpoint	Upper Endpoint	AAPC	Lower CI	Upper CI	Test Statistic~	P-Value~
All ages - 0 Joinpoints	Full Range	1996	2015	2.0	-1.0	5.2	1.4	0.2
45 - 64 years - 0 Joinpoints	Full Range	1996	2015	-1.3	-5.7	3.2	-0.6	0.5
+65 years - 0 Joinpoints	Full Range	1996	2015	1.7	-5.9	10.0	0.5	0.7

* Indicates that the AAPC is significantly different from zero at the alpha = 0.05 level.
~ If the AAPC is within one segment, the t-distribution is used. Otherwise, the normal (z) distribution is used. See Help to Learn More

6) Table. Female incidence with absolute value, age-specific rate, adjusted rate, standard error from 1996 to 2015

VALOR ABSOLUTO

	Total	IGN	0	5	10	15	20	25	30	35	40	45	50	55	60	65	70	75	80	85+
			4	9	14	19	24	29	34	39	44	49	54	59	64	69	74	79	84	
1996	15	0	0	0	0	0	0	0	1	0	0	0	1	2	2	4	2	2	1	0
1997	15	0	0	0	0	0	0	1	0	0	1	2	2	0	2	2	2	2	0	1
1998	29	1	0	0	0	0	2	0	3	0	1	3	1	3	3	1	2	4	3	2
1999	31	0	0	0	0	0	0	0	0	3	0	2	3	4	0	7	0	2	9	1
2000	27	0	0	0	0	0	0	0	2	1	2	1	2	4	2	1	7	3	1	1
2001	25	0	0	0	0	0	0	1	0	1	2	0	2	3	2	1	2	3	2	6
2002	33	0	0	0	0	0	0	0	1	0	0	1	4	4	4	2	9	4	2	2
2003	24	0	0	0	0	1	0	1	1	1	2	4	0	1	3	1	4	1	0	4
2004	27	0	0	0	0	0	0	0	1	0	0	3	1	3	2	1	4	2	6	4
2005	32	0	0	0	0	0	0	0	1	1	2	3	3	2	2	5	1	5	5	2
2006	39	0	0	0	0	0	0	0	1	0	3	5	5	7	3	3	6	2	2	2
2007	31	0	0	0	0	0	0	0	1	0	1	5	5	1	2	3	1	6	3	3
2008	41	0	0	0	0	0	0	1	1	3	2	3	3	2	4	4	5	7	2	4
2009	41	0	0	0	0	0	0	0	2	0	1	4	7	4	3	2	2	7	4	5
2010	46	0	0	0	0	1	0	0	0	1	3	3	6	5	6	3	8	2	1	7
2011	45	0	0	0	0	0	0	0	2	2	2	1	3	7	7	2	3	7	5	4
2012	65	0	0	0	0	1	0	0	0	1	3	5	8	4	7	7	9	8	8	4
2013	59	0	0	0	0	0	0	4	0	3	4	4	4	5	6	10	4	7	5	3
2014	58	0	0	0	0	0	0	0	4	0	2	1	3	5	9	9	7	7	6	5
2015	60	0	0	0	0	0	0	0	2	0	1	3	13	6	5	6	6	7	3	8

TAXA ESPECIFICA

	0	5	10	15	20	25	30	35	40	45	50	55	60	65	70	75	80	85+	Taxa	Taxa
	4	9	14	19	24	29	34	39	44	49	54	59	64	69	74	79	84		Bruta	Ajust.*
1996	0.0	0.0	0.0	0.0	0.0	0.0	5.2	0.0	0.0	0.0	12.7	33.7	40.9	100.1	63.3	100.9	75.4	0.0	6.6	9.6
1997	0.0	0.0	0.0	0.0	0.0	4.6	0.0	0.0	7.2	18.7	25.1	0.0	40.3	49.3	62.3	99.4	0.0	91.3	6.5	9.0
1998	0.0	0.0	0.0	0.0	7.9	0.0	15.1	0.0	7.1	27.7	12.4	49.2	59.7	24.3	61.6	196.3	221.2	180.3	12.4	14.5
1999	0.0	0.0	0.0	0.0	0.0	0.0	0.0	17.7	0.0	18.2	36.7	64.8	0.0	168.3	0.0	96.9	655.5	89.0	13.1	16.3
2000	0.0	0.0	0.0	0.0	0.0	0.0	9.7	5.3	13.0	7.8	20.1	55.3	33.8	22.2	184.8	113.0	57.3	70.7	11.0	12.9
2001	0.0	0.0	0.0	0.0	0.0	4.1	0.0	5.1	12.4	0.0	18.8	39.1	33.1	21.7	52.3	109.0	117.8	435.7	9.8	10.8
2002	0.0	0.0	0.0	0.0	0.0	0.0	4.6	0.0	0.0	7.1	35.7	48.8	64.0	41.2	230.2	140.1	114.7	141.3	12.6	15.5
2003	0.0	0.0	0.0	3.7	0.0	3.8	4.4	4.9	11.2	27.5	0.0	11.4	46.4	19.6	100.3	33.8	0.0	274.2	9.0	10.1
2004	0.0	0.0	0.0	0.0	0.0	0.0	4.3	0.0	0.0	19.9	8.0	32.3	29.7	18.8	97.9	65.3	322.1	264.6	9.8	10.4
2005	0.0	0.0	0.0	0.0	0.0	0.0	4.2	4.7	10.4	19.0	23.1	20.3	28.3	90.7	23.8	158.7	258.3	127.1	11.4	12.1
2006	0.0	0.0	0.0	0.0	0.0	0.0	4.0	0.0	15.1	30.4	37.0	67.2	40.0	52.3	137.9	61.9	99.0	122.0	13.6	15.2
2007	0.0	0.0	0.0	0.0	0.0	0.0	3.9	0.0	4.9	28.7	35.8	9.1	25.1	50.5	22.2	181.8	142.0	175.0	10.6	10.8
2008	0.0	0.0	0.0	0.0	0.0	3.2	3.7	13.1	9.6	16.6	20.7	17.4	47.2	65.0	106.7	207.7	90.4	223.0	13.8	14.2
2009	0.0	0.0	0.0	0.0	0.0	0.0	7.2	0.0	4.7	21.3	46.6	33.2	33.4	31.2	41.1	202.7	172.9	266.2	13.5	13.0
2010	0.0	0.0	0.0	4.0	0.0	0.0	0.0	4.1	13.4	15.3	37.3	38.2	59.8	42.8	150.2	52.0	35.6	270.1	15.0	14.5
2011	0.0	0.0	0.0	0.0	0.0	0.0	6.7	8.0	9.0	5.0	18.3	53.4	70.0	28.1	57.6	190.4	199.5	198.3	14.3	13.5
2012	0.0	0.0	0.0	4.0	0.0	0.0	0.0	3.9	13.3	24.4	46.8	29.4	66.4	92.3	167.8	209.6	309.8	192.4	20.3	19.8
2013	0.0	0.0	0.0	0.0	0.0	12.5	0.0	11.1	17.3	19.2	22.4	35.3	54.1	123.9	72.5	176.5	187.6	139.9	18.1	17.1
2014	0.0	0.0	0.0	0.0	0.0	0.0	12.2	0.0	8.5	4.7	16.1	34.2	75.9	106.9	123.1	169.7	215.5	223.0	17.5	16.3
2015	0	0	0	0	0	6.4	0	3.43	12.6	60.1	31.2	32.8	47.9	67.32	119.2	70.18	103.7	343.3	17.87	17.2

Year	CR	SE
1996	6.6	1.7
1997	6.5	1.7
1998	12.4	2.3
1999	13.1	2.3
2000	11.0	2.1
2001	9.8	2.0
2002	12.6	2.2
2003	9.0	1.8
2004	9.8	1.9
2005	11.4	2.0
2006	13.6	2.2
2007	10.6	1.9
2008	13.8	2.1
2009	13.5	2.1
2010	15.0	2.2
2011	14.3	2.1
2012	20.3	2.5
2013	18.1	2.4
2014	17.5	2.3
2015	17.9	2.3

Year	ASR	SE
1996	9.6	2.5
1997	9.0	2.3
1998	14.5	2.7
1999	16.3	2.9
2000	12.9	2.5
2001	10.8	2.2
2002	15.5	2.7
2003	10.1	2.1
2004	10.4	2.0
2005	12.1	2.1
2006	15.2	2.4
2007	10.8	1.9
2008	14.2	2.2
2009	13.0	2.0
2010	14.5	2.1
2011	13.5	2.0
2012	19.8	2.5
2013	17.1	2.2
2014	16.3	2.1
2015	17.2	2.2

Year	0-19	SE
1996	0.0	#DIV/0!
1997	0.0	#DIV/0!
1998	0.0	#DIV/0!
1999	0.0	#DIV/0!
2000	0.0	#DIV/0!
2001	0.0	#DIV/0!
2002	0.0	#DIV/0!
2003	0.9	0.9
2004	0.0	#DIV/0!
2005	0.0	#DIV/0!
2006	0.0	#DIV/0!
2007	0.0	#DIV/0!
2008	0.0	#DIV/0!
2009	0.0	#DIV/0!
2010	1.0	1.0
2011	0.0	#DIV/0!
2012	1.0	1.0
2013	0.0	#DIV/0!
2014	0.0	#DIV/0!
2015	0.0	#DIV/0!

Year	20-44	SE
1996	1.0	1.0
1997	2.4	1.7
1998	6.0	2.5
1999	3.5	2.0
2000	5.6	2.5
2001	4.3	2.2
2002	0.9	0.9
2003	4.9	2.2
2004	0.9	0.9
2005	3.8	1.9
2006	3.8	1.9
2007	1.8	1.2
2008	5.9	2.2
2009	2.4	1.4
2010	3.5	1.8
2011	4.7	1.9
2012	3.4	1.7
2013	8.2	2.5
2014	4.1	1.7
2015	4.5	2.6

Year	45-64	SE
1996	21.8	9.8
1997	21.0	8.6
1998	37.2	11.8
1999	29.9	10.0
2000	29.3	9.8
2001	22.8	8.6
2002	38.9	10.8
2003	21.3	7.5
2004	22.5	7.5
2005	22.7	7.2
2006	43.7	9.8
2007	24.7	6.8
2008	25.5	7.4
2009	33.6	7.9
2010	37.7	8.4
2011	36.7	8.6
2012	41.8	8.5
2013	32.8	7.5
2014	32.7	7.7
2015	43.0	8.3

Year	65+	SE
1996	67.9	22.6
1997	60.5	22.9
1998	136.7	39.5
1999	202.0	46.3
2000	89.6	24.8
2001	147.3	39.4
2002	133.5	30.6
2003	85.6	27.1
2004	153.7	37.3
2005	131.7	31.0
2006	94.6	24.4
2007	114.3	28.6
2008	138.5	29.5
2009	142.8	31.9
2010	110.1	24.0
2011	134.8	29.4
2012	194.4	32.4
2013	140.1	26.0
2014	167.6	28.7
2015	140.7	25.7

7) Table. Male incidence with absolute value, age-specific rate, adjusted rate, standard error from 1996 to 2015

			VALOR ABSOLUTO																		TAXA ESPECIFICA																			
	Total	IGN	0	5	10	15	20	25	30	35	40	45	50	55	60	65	70	75	80	85+	0	5	10	15	20	25	30	35	40	45	50	55	60	65	70	75	80	85+	Taxa	Taxa
			4	9	14	19	24	29	34	39	44	49	54	59	64	69	74	79	84		4	9	14	19	24	29	34	39	44	49	54	59	64	69	74	79	84		Bruta	Ajust.*
1996	10	0	0	0	0	0	0	0	1	0	0	0	2	1	1	2	1	2	0	0	0.0	0.0	0.0	0.0	0.0	0.0	6.2	0.0	0.0	0.0	29.8	21.2	29.0	77.8	51.4	165.3	0.0	0.0	5.0	8.9
1997	14	0	0	0	0	0	0	0	0	1	0	0	2	1	2	3	1	0	2	1	0.0	0.0	0.0	0.0	0.0	0.0	0.0	7.1	0.0	0.0	29.3	20.8	57.1	114.9	50.7	0.0	252.5	197.2	6.9	11.7
1998	14	0	0	0	0	0	0	0	0	0	2	1	3	1	2	3	1	1	0	0	0.0	0.0	0.0	0.0	0.0	0.0	0.0	0.0	16.7	10.7	43.5	20.6	56.4	113.5	50.1	80.4	0.0	0.0	6.8	12.1
1999	14	0	0	0	0	0	0	1	1	0	0	1	1	1	1	0	2	1	3	2	0.0	0.0	0.0	0.0	0.0	5.2	6.0	0.0	0.0	10.5	14.3	20.3	27.9	0.0	98.9	79.4	369.5	385.4	6.7	10.6
2000	15	0	0	0	0	0	0	0	0	1	1	1	0	2	3	1	4	1	0	1	0.0	0.0	0.0	0.0	0.0	0.0	0.0	6.5	7.7	9.4	0.0	34.4	70.4	34.6	182.7	68.5	0.0	143.3	6.9	11.7
2001	19	0	0	0	0	0	1	0	1	1	0	1	4	2	3	2	2	1	1	0	0.0	0.0	0.0	0.0	4.0	0.0	5.3	6.0	0.0	8.7	44.6	32.2	68.7	67.0	89.7	6.9	117.2	0.0	8.3	12.2
2002	18	0	0	0	0	0	0	1	0	0	1	1	2	3	5	1	2	0	0	2	0.0	0.0	0.0	0.0	0.0	4.5	0.0	0.0	6.8	8.4	21.3	45.0	110.0	31.7	87.2	0.0	0.0	327.9	7.7	12.9
2003	18	0	0	0	0	0	0	0	0	1	2	0	1	3	3	3	2	2	0	1	0.0	0.0	0.0	0.0	0.0	0.0	0.0	5.7	13.1	0.0	10.2	42.0	63.1	91.0	84.8	132.9	0.0	160.8	7.5	12.4
2004	14	0	0	0	1	0	0	0	0	0	1	3	1	1	2	2	2	1	0	0	0.0	0.0	4.4	0.0	0.0	0.0	0.0	0.0	6.3	23.1	9.8	13.2	40.0	58.2	82.2	65.2	0.0	0.0	5.7	8.8
2005	22	0	0	0	0	0	0	1	0	0	0	3	2	0	5	4	3	2	1	1	0.0	0.0	0.0	0.0	0.0	3.9	0.0	0.0	0.0	22.2	18.7	0.0	94.2	111.9	119.0	127.9	109.3	154.8	8.7	14.7
2006	31	0	0	0	0	0	0	0	0	2	0	3	5	5	1	4	3	2	2	4	0.0	0.0	0.0	0.0	0.0	0.0	0.0	10.5	0.0	21.3	44.9	59.3	17.6	106.0	114.3	125.1	214.1	606.1	12.1	18.1
2007	31	0	0	0	0	0	1	0	0	1	2	0	3	5	3	5	4	2	4	1	0.0	0.0	0.0	0.0	3.5	0.0	0.0	5.2	11.6	0.0	25.9	56.8	49.5	126.2	146.3	122.9	418.0	147.9	11.8	17.6
2008	27	0	0	0	0	0	0	0	0	5	0	2	0	1	4	4	2	6	1	2	0.0	0.0	0.0	0.0	0.0	0.0	0.0	25.2	0.0	13.2	0.0	10.9	61.9	96.2	70.0	361.0	101.8	288.6	10.1	15.1
2009	34	0	0	0	0	0	0	0	1	3	3	2	1	4	5	3	6	3	1	2	0.0	0.0	0.0	0.0	0.0	0.0	4.1	14.7	16.6	12.8	7.9	42.0	73.0	68.7	200.6	176.3	99.1	280.9	12.5	17.6
2010	32	0	0	0	0	0	0	0	0	2	1	1	4	4	4	4	6	1	2	3	0.0	0.0	0.0	0.0	0.0	0.0	0.0	9.9	5.5	6.3	30.8	40.0	52.9	80.3	175.6	50.2	145.0	315.5	12.1	15.3
2011	32	0	0	0	0	0	0	2	0	1	1	4	1	4	5	3	2	3	2	4	0.0	0.0	0.0	0.0	0.0	6.7	0.0	4.6	5.3	24.1	7.3	38.6	65.7	60.9	64.2	167.3	189.8	546.4	11.4	15.6
2012	45	0	0	0	0	0	0	0	0	3	1	9	4	4	3	6	6	4	1	4	0.0	0.0	0.0	0.0	0.0	0.0	0.0	13.3	5.2	53.2	28.5	36.9	37.6	113.6	186.0	217.2	93.9	540.5	15.8	21.2
2013	35	0	0	0	0	0	0	1	0	3	3	3	0	6	5	5	4	5	0	0	0.0	0.0	0.0	0.0	0.0	3.4	0.0	12.8	15.2	17.4	0.0	53.1	60.0	88.3	119.7	263.3	0.0	0.0	12.1	15.2
2014	41	0	0	0	0	0	0	1	1	2	2	2	3	4	7	10	4	1	2	2	0.0	0.0	0.0	0.0	0.0	3.4	3.4	8.1	10.0	11.4	19.9	34.0	80.0	167.9	115.0	50.7	180.0	259.1	14.0	17.8
2015	42	0	0	0	0	0	0	1	0	3	5	2	3	2	6	6	5	4	2	3	0	0	0	0	4	0	10	19	10	17	13	49	65	79	111	97.8	264	0	14	15.8

Year	CR	SE
1996	5.0	1.6
1997	6.9	1.8
1998	6.8	1.8
1999	6.7	1.8
2000	6.9	1.8
2001	8.3	1.9
2002	7.7	1.8
2003	7.5	1.8
2004	5.7	1.5
2005	8.7	1.9
2006	12.1	2.2
2007	11.8	2.1
2008	10.1	1.9
2009	12.5	2.1
2010	12.1	2.1
2011	11.4	2.0
2012	15.8	2.4
2013	12.1	2.0
2014	14.0	2.2
2015	14.1	2.2

Year	ASR	SE
1996	8.9	2.8
1997	11.7	3.1
1998	12.1	3.2
1999	10.6	2.8
2000	11.7	3.0
2001	12.2	2.8
2002	12.9	3.0
2003	12.4	2.9
2004	8.8	2.4
2005	14.7	3.1
2006	18.1	3.2
2007	17.6	3.2
2008	15.1	2.9
2009	17.6	3.0
2010	15.3	2.7
2011	15.6	2.8
2012	21.2	3.2
2013	15.2	2.6
2014	17.8	2.8
2015	15.8	2.4

Year	0-19	SE
1996	0.0	#DIV/0!
1997	0.0	#DIV/0!
1998	0.0	#DIV/0!
1999	0.0	#DIV/0!
2000	0.0	#DIV/0!
2001	0.0	#DIV/0!
2002	0.0	#DIV/0!
2003	0.0	#DIV/0!
2004	1.1	1.1
2005	0.0	#DIV/0!
2006	0.0	#DIV/0!
2007	0.0	#DIV/0!
2008	0.0	#DIV/0!
2009	0.0	#DIV/0!
2010	0.0	#DIV/0!
2011	0.0	#DIV/0!
2012	0.0	#DIV/0!
2013	0.0	#DIV/0!
2014	0.0	#DIV/0!
2015	0.0	#DIV/0!

Year	20-44	SE
1996	1.2	1.2
1997	1.4	1.4
1998	3.3	2.4
1999	2.3	1.6
2000	2.8	2.0
2001	3.1	1.8
2002	2.3	1.6
2003	3.8	2.2
2004	1.3	1.3
2005	0.8	0.8
2006	2.1	1.5
2007	4.1	2.0
2008	5.0	2.3
2009	7.1	2.7
2010	3.1	1.8
2011	3.3	1.7
2012	3.7	1.8
2013	6.3	2.4
2014	5.0	2.0
2015	8.7	2.9

Year	45-64	SE
1996	20.0	10.0
1997	26.8	12.0
1998	32.8	12.4
1999	18.3	9.1
2000	28.6	11.7
2001	38.6	12.2
2002	46.2	13.9
2003	28.8	10.9
2004	21.5	8.1
2005	33.8	10.7
2006	35.8	9.6
2007	33.1	10.0
2008	21.5	8.1
2009	33.9	9.8
2010	32.5	9.0
2011	33.9	9.1
2012	39.1	8.7
2013	32.6	8.7
2014	36.3	9.1
2015	36.0	10.0

Year	65+	SE
1996	58.9	26.3
1997	123.1	46.5
1998	48.8	21.8
1999	186.6	66.0
2000	85.8	32.4
2001	56.2	22.9
2002	89.4	40.0
2003	93.9	33.2
2004	41.1	18.4
2005	124.6	37.6
2006	233.1	60.2
2007	192.2	48.1
2008	183.5	47.4
2009	165.1	42.6
2010	153.3	38.3
2011	205.7	55.0
2012	230.2	50.2
2013	94.3	25.2
2014	154.5	35.5
2015	110.5	24.7

8) Table. Female mortality with absolute value, age-specific rate, adjusted rate, standard error from 1996 to 2015

	VALOR ABSOLUTO																			
	Total	IGN	0 4	5 9	10 14	15 19	20 24	25 29	30 34	35 39	40 44	45 49	50 54	55 59	60 64	65 69	70 74	75 79	80 84	85+
1996	3	0	0	0	0	0	0	0	0	0	0	0	0	0	1	1	0	1	0	0
1997	7	1	0	0	0	0	0	0	0	0	0	1	0	0	1	0	1	2	0	1
1998	7	1	0	0	0	0	0	1	0	0	0	0	1	1	0	1	0	1	1	0
1999	15	0	0	0	0	0	1	0	0	0	0	1	2	1	1	3	2	1	3	0
2000	13	0	0	0	0	0	0	0	0	1	0	1	0	0	0	1	3	2	3	2
2001	9	0	0	0	0	0	0	0	0	0	1	1	0	1	0	0	1	2	0	3
2002	8	0	0	0	0	0	0	0	0	0	0	0	0	2	1	1	1	1	1	1
2003	7	0	0	0	0	0	0	0	0	0	1	1	0	0	1	0	1	0	0	3
2004	13	0	0	0	0	0	0	0	1	0	0	1	0	1	1	0	4	0	4	1
2005	17	0	0	0	0	0	0	0	1	0	0	3	1	3	1	1	0	2	4	1
2006	14	0	0	0	0	0	0	0	0	0	0	0	1	3	2	2	3	2	1	0
2007	14	0	0	0	0	0	0	0	1	0	1	1	4	2	0	2	0	1	0	2
2008	23	0	0	0	0	0	0	0	0	0	0	1	1	2	3	2	3	5	1	5
2009	13	0	0	0	0	0	0	0	0	0	0	0	1	0	1	1	1	6	1	2
2010	18	0	0	0	0	0	0	0	0	0	0	1	2	6	0	1	3	2	1	2
2011	13	0	0	0	0	0	0	0	0	0	0	0	3	2	0	1	0	4	0	3
2012	22	0	0	0	0	0	0	0	0	0	0	0	2	1	2	0	3	4	4	6
2013	20	0	0	0	0	0	0	0	0	0	0	0	3	1	5	2	3	3	2	1
2014	14	0	0	0	0	0	1	0	0	2	1	1	0	3	2	1	1	2	0	0
2015	18	0	0	0	0	0	0	0	0	0	1	2	0	0	5	3	2	2	1	2

	TAXA ESPECIFICA																			
	0 4	5 9	10 14	15 19	20 24	25 29	30 34	35 39	40 44	45 49	50 54	55 59	60 64	65 69	70 74	75 79	80 84	85+	Taxa Bruta	Taxa Ajust.*
1996	0.0	0.0	0.0	0.0	0.0	0.0	0.0	0.0	0.0	0.0	0.0	0.0	20.4	25.0	0.0	50.4	0.0	0.0	1.3	2.1
1997	0.0	0.0	0.0	0.0	0.0	0.0	0.0	0.0	0.0	9.4	0.0	0.0	20.1	0.0	31.2	99.4	0.0	91.3	3.0	3.4
1998	0.0	0.0	0.0	0.0	0.0	4.5	0.0	0.0	0.0	0.0	12.4	16.4	0.0	24.3	0.0	49.1	73.7	0.0	3.0	3.2
1999	0.0	0.0	0.0	0.0	3.9	0.0	0.0	0.0	0.0	9.1	24.5	16.2	19.7	72.1	60.8	48.5	218.5	0.0	6.3	8.5
2000	0.0	0.0	0.0	0.0	0.0	0.0	0.0	5.3	0.0	7.8	0.0	0.0	0.0	22.2	79.2	75.3	172.0	141.3	5.3	5.4
2001	0.0	0.0	0.0	0.0	0.0	0.0	0.0	0.0	6.2	7.4	0.0	13.0	0.0	0.0	26.1	72.6	0.0	217.9	3.5	3.7
2002	0.0	0.0	0.0	0.0	0.0	0.0	0.0	0.0	0.0	0.0	0.0	24.4	16.0	20.6	25.6	35.0	57.4	70.7	3.1	3.7
2003	0.0	0.0	0.0	0.0	0.0	0.0	0.0	0.0	5.6	6.9	0.0	0.0	15.5	0.0	25.1	0.0	0.0	205.6	2.6	2.9
2004	0.0	0.0	0.0	0.0	0.0	0.0	4.3	0.0	0.0	6.6	0.0	10.8	14.8	0.0	97.9	0.0	214.7	66.1	4.7	5.0
2005	0.0	0.0	0.0	0.0	0.0	0.0	4.2	0.0	0.0	19.0	7.7	30.5	14.2	18.1	0.0	63.5	206.6	63.6	6.1	6.1
2006	0.0	0.0	0.0	0.0	0.0	0.0	0.0	0.0	0.0	0.0	7.4	28.8	26.7	34.9	68.9	61.9	49.5	0.0	4.9	5.9
2007		0.0	0.0	0.0	0.0	0.0	3.9	0.0	4.9	5.7	28.6	18.2	0.0	33.6	0.0	30.3	0.0	116.7	4.8	4.9
2008	0.0	0.0	0.0	0.0	0.0	0.0	0.0	0.0	0.0	5.5	6.9	17.4	35.4	32.5	64.0	148.3	45.2	278.7	7.7	8.1
2009	0.0	0.0	0.0	0.0	0.0	0.0	0.0	0.0	0.0	0.0	6.7	0.0	11.1	15.6	20.6	173.8	43.2	106.5	4.3	4.1
2010	0.0	0.0	0.0	0.0	0.0	0.0	0.0	0.0	0.0	5.1	12.4	45.9	0.0	14.3	56.3	52.0	35.6	77.2	5.9	5.4
2011	0.0	0.0	0.0	0.0	0.0	0.0	0.0	0.0	0.0	0.0	18.3	15.3	0.0	14.0	0.0	108.8	0.0	148.7	4.1	3.8
2012	0.0	0.0	0.0	0.0	0.0	0.0	0.0	0.0	0.0	0.0	11.7	7.3	19.0	0.0	55.9	104.8	154.9	288.6	6.9	6.0
2013	0.0	0.0	0.0	0.0	0.0	0.0	0.0	0.0	0.0	0.0	16.8	7.1	45.1	24.8	54.4	75.6	75.0	46.6	6.1	6.1
2014	0.0	0.0	0.0	0.0	3.6	0.0	0.0	7.2	4.3	4.7	0.0	20.5	16.9	11.9	17.6	48.5	0.0	0.0	4.2	3.9
2015	0.0	0.0	0.0	0.0	0.0	0.0	0.0	3.4	8.4	0.0	0.0	32.8	24.0	22.4	34.1	23.4	34.6	85.8	5.4	5.2

Year	CR	SE
1996	1.3	0.8
1997	3.0	1.1
1998	3.0	1.1
1999	6.3	1.6
2000	5.3	1.5
2001	3.5	1.2
2002	3.1	1.1
2003	2.6	1.0
2004	4.7	1.3
2005	6.1	1.5
2006	4.9	1.3
2007	4.8	1.3
2008	7.7	1.6
2009	4.3	1.2
2010	5.9	1.4
2011	4.1	1.1
2012	6.9	1.5
2013	6.1	1.4
2014	4.2	1.1
2015	5.4	1.3

Year	ASR	SE
1996	2.1	1.2
1997	3.4	1.3
1998	3.2	1.2
1999	8.5	2.2
2000	5.4	1.5
2001	3.7	1.2
2002	3.7	1.3
2003	2.9	1.1
2004	5.0	1.4
2005	6.1	1.5
2006	5.9	1.6
2007	4.9	1.3
2008	8.1	1.7
2009	4.1	1.1
2010	5.4	1.3
2011	3.8	1.0
2012	6.0	1.3
2013	6.1	1.4
2014	3.9	1.1
2015	5.2	1.2

Year	0-19	SE
1996	0.0	#DIV/0!
1997	0.0	#DIV/0!
1998	0.0	#DIV/0!
1999	0.0	#DIV/0!
2000	0.0	#DIV/0!
2001	0.0	#DIV/0!
2002	0.0	#DIV/0!
2003	0.0	#DIV/0!
2004	0.0	#DIV/0!
2005	0.0	#DIV/0!
2006	0.0	#DIV/0!
2007	0.0	#DIV/0!
2008	0.0	#DIV/0!
2009	0.0	#DIV/0!
2010	0.0	#DIV/0!
2011	0.0	#DIV/0!
2012	0.0	#DIV/0!
2013	0.0	#DIV/0!
2014	0.0	#DIV/0!
2015	0.0	#DIV/0!

Year	20-44	SE
1996	0.0	#DIV/0!
1997	0.0	#DIV/0!
1998	0.9	0.9
1999	0.8	0.8
2000	1.1	1.1
2001	1.2	1.2
2002	0.0	#DIV/0!
2003	1.1	1.1
2004	0.9	0.9
2005	0.8	0.8
2006	0.0	#DIV/0!
2007	1.8	1.2
2008	0.0	#DIV/0!
2009	0.0	#DIV/0!
2010	0.0	#DIV/0!
2011	0.0	#DIV/0!
2012	0.0	#DIV/0!
2013	0.0	#DIV/0!
2014	3.0	1.5
2015	2.4	2.4

Year	45-64	SE
1996	5.1	5.1
1997	7.4	5.2
1998	7.2	5.1
1999	17.4	7.8
2000	1.9	1.9
2001	5.1	3.6
2002	10.1	5.8
2003	5.6	3.9
2004	8.1	4.6
2005	17.8	6.3
2006	15.7	6.4
2007	13.1	5.0
2008	16.3	6.2
2009	4.4	3.1
2010	15.9	5.3
2011	8.4	3.8
2012	9.5	4.3
2013	17.3	5.8
2014	10.5	4.3
2015	14.2	5.4

Year	65+	SE
1996	15.1	10.7
1997	44.4	22.2
1998	29.4	17.0
1999	80.0	26.7
2000	98.0	29.6
2001	63.3	25.9
2002	41.9	18.7
2003	46.1	23.1
2004	75.7	25.2
2005	70.4	24.9
2006	43.0	15.2
2007	36.1	16.2
2008	113.8	28.4
2009	71.9	21.7
2010	47.1	15.7
2011	54.3	19.2
2012	120.9	29.3
2013	55.3	16.7
2014	15.6	7.8
2015	40.1	12.7

9) Table. Male mortality with absolute value, age-specific rate, adjusted rate, standard error from 1996 to 2015

			VALOR ABSOLUTO																		TAXA ESPECIFICA																			
	Total	IGN	0	5	10	15	20	25	30	35	40	45	50	55	60	65	70	75	80	85+	0	5	10	15	20	25	30	35	40	45	50	55	60	65	70	75	80	85+	Taxa	Taxa
			4	9	14	19	24	29	34	39	44	49	54	59	64	69	74	79	84		4	9	14	19	24	29	34	39	44	49	54	59	64	69	74	79	84		Bruta	Ajust.*
1996	3	0	0	0	0	0	0	0	1	0	0	0	1	0	0	1	0	0	0	0	0.0	0.0	0.0	0.0	0.0	0.0	6.2	0.0	0.0	0.0	14.9	0.0	0.0	38.9	0.0	0.0	0.0	0.0	1.5	2.3
1997	4	0	0	0	0	0	0	0	0	0	0	1	0	0	0	1	1	1	0	0	0.0	0.0	0.0	0.0	0.0	0.0	0.0	0.0	0.0	10.8	0.0	0.0	0.0	38.3	50.7	81.4	0.0	0.0	2.0	3.6
1998	9	0	1	0	0	0	0	0	0	0	0	2	0	3	1	1	0	1	0	0	5.1	0.0	0.0	0.0	0.0	0.0	0.0	0.0	0.0	21.4	0.0	61.8	28.2	37.8	0.0	80.4	0.0	0.0	4.4	7.4
1999	8	0	0	0	0	0	0	0	1	0	0	0	0	1	1	0	2	2	1	0	0.0	0.0	0.0	0.0	0.0	0.0	6.0	0.0	0.0	0.0	0.0	20.3	27.9	0.0	98.9	158.9	123.2	0.0	3.8	6.5
2000	3	0	0	0	0	0	0	0	0	0	0	1	0	1	1	0	0	0	0	0	0.0	0.0	0.0	0.0	0.0	0.0	0.0	0.0	0.0	9.4	0.0	17.2	23.5	0.0	0.0	0.0	0.0	0.0	1.4	2.2
2001	7	0	0	0	0	0	0	0	0	0	0	0	0	2	3	0	2	0	0	0	0.0	0.0	0.0	0.0	0.0	0.0	0.0	0.0	0.0	0.0	0.0	32.2	68.7	0.0	89.7	0.0	0.0	0.0	3.1	5.8
2002	5	0	0	0	0	0	0	0	0	0	0	0	1	1	2	0	0	0	1	0	0.0	0.0	0.0	0.0	0.0	0.0	0.0	0.0	0.0	0.0	10.6	15.0	44.0	0.0	0.0	0.0	115.6	0.0	2.1	3.5
2003	10	0	0	0	0	0	0	0	0	1	2	0	1	2	2	1	1	0	0	0	0.0	0.0	0.0	0.0	0.0	0.0	0.0	5.7	13.1	0.0	10.2	28.0	42.0	30.3	42.4	0.0	0.0	0.0	4.2	6.2
2004	3	0	0	0	0	0	0	0	0	0	0	0	1	0	0	2	0	0	0	0	0.0	0.0	0.0	0.0	0.0	0.0	0.0	0.0	0.0	0.0	9.8	0.0	0.0	58.2	0.0	0.0	0.0	0.0	1.2	2.2
2005	11	0	0	0	0	0	0	0	0	0	1	1	0	1	1	1	1	3	1	1	0.0	0.0	0.0	0.0	0.0	0.0	0.0	0.0	6.1	7.4	0.0	12.5	18.8	28.0	39.7	191.8	109.3	154.8	4.4	6.9
2006	12	0	0	0	0	0	0	0	0	0	0	0	1	1	1	3	1	2	1	2	0.0	0.0	0.0	0.0	0.0	0.0	0.0	0.0	0.0	0.0	9.0	11.9	17.6	79.5	38.1	125.1	107.1	303.0	4.7	8.1
2007	10	0	0	0	0	0	1	1	0	0	0	0	1	1	1	1	2	1	0	1	0.0	0.0	0.0	0.0	3.5	3.7	0.0	0.0	0.0	0.0	8.6	11.4	16.5	25.2	73.2	61.4	0.0	147.9	3.8	5.7
2008	14	0	0	0	0	0	0	0	0	1	0	0	0	2	0	0	4	2	3	2	0.0	0.0	0.0	0.0	0.0	0.0	0.0	5.0	0.0	0.0	0.0	21.8	0.0	0.0	140.0	120.3	305.5	288.6	5.2	8.1
2009	16	0	0	0	0	0	0	0	0	1	0	0	2	2	3	1	3	2	0	2	0.0	0.0	0.0	0.0	0.0	0.0	0.0	4.9	0.0	0.0	15.9	21.0	43.8	22.9	100.3	117.5	0.0	280.9	5.9	9.0
2010	13	0	0	0	0	0	0	0	0	0	1	1	3	1	1	2	2	0	0	2	0.0	0.0	0.0	0.0	0.0	0.0	0.0	0.0	5.5	6.3	23.1	10.0	13.2	40.1	58.5	0.0	0.0	210.3	4.9	6.2
2011	14	0	0	0	0	0	0	0	0	1	0	1	2	3	1	2	0	1	0	3	0.0	0.0	0.0	0.0	0.0	0.0	0.0	4.6	0.0	6.0	14.7	28.9	13.1	40.6	0.0	55.8	0.0	409.8	5.0	6.9
2012	19	0	0	0	0	0	0	0	0	1	1	3	1	2	1	3	3	2	0	2	0.0	0.0	0.0	0.0	0.0	0.0	0.0	4.4	5.2	17.7	7.1	18.5	12.5	56.8	93.0	108.6	0.0	270.3	6.7	9.2
2013	11	0	0	0	0	0	0	1	0	0	1	1	0	1	2	1	0	1	1	2	0.0	0.0	0.0	0.0	0.0	3.4	0.0	0.0	5.1	5.8	0.0	8.8	24.0	17.7	0.0	52.7	92.7	266.7	3.8	5.1
2014	9	0	0	0	0	0	0	0	1	0	1	1	2	1	1	0	0	0	0	2	0.0	0.0	0.0	0.0	0.0	0.0	3.4	0.0	5.0	5.7	13.3	8.5	11.4	0.0	0.0	0.0	0.0	259.1	3.1	3.6
2015	18	0	0	0	0	0	0	1	1	1	1	0	0	0	2	7	2	3	0	0	0	0	0	0	4	3	3	4	0	0	0	16	76	32	83.1	0	0	0	6.06	7.347

Year	CR	SE
1996	1.5	0.9
1997	2.0	1.0
1998	4.4	1.5
1999	3.8	1.4
2000	1.4	0.8
2001	3.1	1.2
2002	2.1	1.0
2003	4.2	1.3
2004	1.2	0.7
2005	4.4	1.3
2006	4.7	1.3
2007	3.8	1.2
2008	5.2	1.4
2009	5.9	1.5
2010	4.9	1.4
2011	5.0	1.3
2012	6.7	1.5
2013	3.8	1.1
2014	3.1	1.0
2015	6.1	1.4

Year	ASR	SE
1996	2.3	1.3
1997	3.6	1.8
1998	7.4	2.5
1999	6.5	2.3
2000	2.2	1.3
2001	5.8	2.2
2002	3.5	1.6
2003	6.2	2.0
2004	2.2	1.3
2005	6.9	2.1
2006	8.1	2.3
2007	5.7	1.8
2008	8.1	2.2
2009	9.0	2.2
2010	6.2	1.7
2011	6.9	1.8
2012	9.2	2.1
2013	5.1	1.5
2014	3.6	1.2
2015	7.3	1.7

Year	0-19	SE
1996	0.0	#DIV/0!
1997	0.0	#DIV/0!
1998	1.3	1.3
1999	0.0	#DIV/0!
2000	0.0	#DIV/0!
2001	0.0	#DIV/0!
2002	0.0	#DIV/0!
2003	0.0	#DIV/0!
2004	0.0	#DIV/0!
2005	0.0	#DIV/0!
2006	0.0	#DIV/0!
2007	0.0	#DIV/0!
2008	0.0	#DIV/0!
2009	0.0	#DIV/0!
2010	0.0	#DIV/0!
2011	0.0	#DIV/0!
2012	0.0	#DIV/0!
2013	0.0	#DIV/0!
2014	0.0	#DIV/0!
2015	0.0	#DIV/0!

Year	20-44	SE
1996	1.2	1.2
1997	0.0	#DIV/0!
1998	0.0	#DIV/0!
1999	1.2	1.2
2000	0.0	#DIV/0!
2001	0.0	#DIV/0!
2002	0.0	#DIV/0!
2003	3.8	2.2
2004	0.0	#DIV/0!
2005	1.2	1.2
2006	0.0	#DIV/0!
2007	1.4	1.0
2008	1.0	1.0
2009	1.0	1.0
2010	1.1	1.1
2011	0.9	0.9
2012	1.9	1.4
2013	1.7	1.2
2014	1.7	1.2
2015	2.9	1.5

Year	45-64	SE
1996	3.7	3.7
1997	2.7	2.7
1998	27.8	11.4
1999	12.0	8.5
2000	12.5	7.2
2001	25.2	11.3
2002	17.4	8.7
2003	20.1	9.0
2004	2.4	2.4
2005	9.7	5.6
2006	9.6	5.6
2007	9.1	5.3
2008	5.5	3.9
2009	20.2	7.6
2010	13.2	5.4
2011	15.7	5.9
2012	14.0	5.3
2013	9.7	4.8
2014	9.7	4.3
2015	23.1	16.4

Year	65+	SE
1996	7.8	7.8
1997	34.1	19.7
1998	23.6	16.7
1999	76.2	34.1
2000	0.1	0.3
2001	17.9	12.7
2002	23.1	23.1
2003	14.5	10.3
2004	11.6	8.2
2005	104.7	39.6
2006	130.6	43.5
2007	61.5	27.5
2008	170.9	51.5
2009	104.3	36.9
2010	61.8	25.2
2011	101.2	41.3
2012	105.7	33.4
2013	85.9	38.4
2014	51.8	36.6
2015	23.0	6.6
